# DNA double-strand break signaling and human disorders

**DOI:** 10.1186/2041-9414-1-15

**Published:** 2010-11-05

**Authors:** Toshiyuki Bohgaki, Miyuki Bohgaki, Razqallah Hakem

**Affiliations:** 1 Ontario Cancer Institute, University Health Network and Department of Medical Biophysics, University of Toronto, 610 University Avenue, Toronto, M5G 2M9 Ontario, Canada

## Abstract

DNA double-strand breaks are among the most serious types of DNA damage and their signaling and repair is critical for all cells and organisms. The repair of both induced and programmed DNA breaks is fundamental as demonstrated by the many human syndromes, neurodegenerative diseases, immunodeficiency and cancer associated with defective repair of these DNA lesions. Homologous recombination and non-homologous end-joining pathways are the two major DNA repair pathways responsible for mediating the repair of DNA double-strand breaks. The signaling of DNA double-strand breaks is critical for cells to orchestrate the repair pathways and maintain genomic integrity. This signaling network is highly regulated and involves a growing number of proteins and elaborated posttranslational modifications including phosphorylation and ubiquitylation. Here, we highlight the recent progress in the signaling of DNA double-strand breaks, the major proteins and posttranslational modifications involved and the diseases and syndromes associated with impaired signaling of these breaks.

## Background

Mammalian cells and organisms have evolved elegant ways to maintain their genomic integrity and respond to the various DNA lesions that they continuously face. DNA damage can result from exogenous stresses, such as ionizing radiation (IR), ultraviolet (UV) light and chemical compounds, or from endogenous insults such as reactive oxygen species (ROS) and DNA replication errors [1].

DNA double-strand breaks (DSBs) are among the most serious and lethal types of DNA damage, as a single DSB is sufficient to kill a cell or disturb its genomic integrity [1]. DSBs are generated in response to exogenous and endogenous DNA insults. For instance, DSBs are induced in response to oncogenic activation [2]. In human precancerous lesions, oncogene activation has been shown to lead to continuous formation of DNA DSBs [3,4]. These DSBs activate the tumor suppressor p53 that mediate apoptosis and/or senescence to restrain the growth of the precancerous cells. In the presence of additional mutations that inactivate p53, precancerous cells become cancerous as they escape p53 mediated apoptosis and/or senescence [5,6]. In addition to the induced DSBs, there are also programmed DSBs that are critical for physiological processes such as meiosis and T and B-cell receptor rearrangements [7,8].

**Figure 1:**
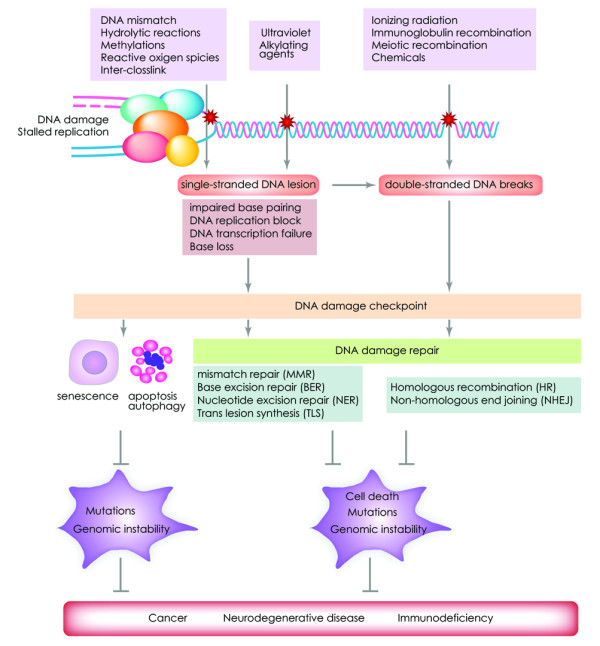
**Mammalian DNA damage repair pathways and checkpoints**. Various exogenous and endogenous sources can generate damaged DNA. In response to DNA damage cells activate the appropriate DNA damage repair and checkpoint pathways or apoptosis. Lesions of single or double-stranded DNA lead to the activation of cell cycle checkpoints and a number of DNA damage repair pathways including MMR (mismatch repair), BER (base excision repair), NER (nucleotide excision repair), TLS (translesion DNA synthesis), HR (homologous recombination) and NHEJ (non-homologous end joining). Impaired repair of damaged DNA can lead to accumulation of, mutations and genomic instability. In addition, defective repair of damaged DNA can lead to aging as well as predisposition for various genetic diseases including cancer and immunodeficiency.

**Figure 2:**
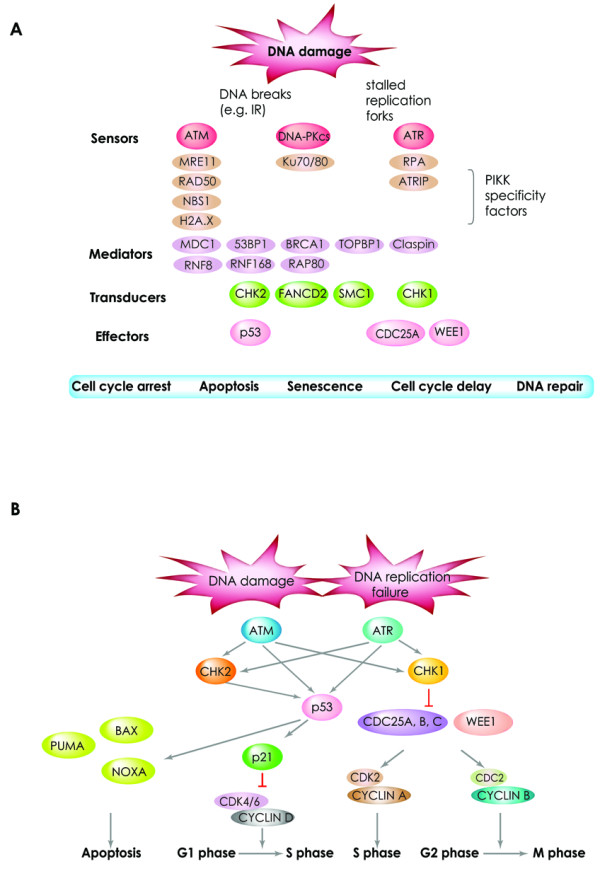
**Schematic representation of the DNA damage-signaling that leads to activation of cell cycle checkpoints or apoptosis**. **(A) **Examples of proteins involved in the different steps of DNA damage signaling are shown. DNA lesions are recognized by sensors (e.g ATM), and mediators (e.g 53BP1) serve to amplify the signaling of DNA damage. Next, proteins including CHK2 serve to transduce the DNA damage signals. Finally, effectors (e.g p53) are required to trigger the appropriate DNA damage cellular responses that include apoptosis, senescence or cell cycle arrest or delay that allow cells to repair their damaged DNA. **(B) **In response to DSBs and/or DNA replication failure, activated ATM and/or ATR phosphorylate the CHK2 and CHK1. Activated ATM, ATR and CHK2 also phosphorylate p53, thus increasing its stability and activation. Activated p53 transactivates the *p21 *that inhibits the cyclin-dependent-kinases and delays the G1/S transition. In the case the damage DNA is beyond repair, p53 can promote apoptosis of the damaged cells through the transactivation of its transcriptional targets including the *Bax*, *Puma *and *Noxa*. CHK1 is essential for S and G2/M checkpoints activation. CDC25C inactivation and WEE1 activation through their phosphorylation by CHK1 result in the inhibition of CDC2/cyclin B activity and G2/M arrest. CDC25C dephosphorylates CDC2 leading to its activation [177]. In response to DNA damage, CHK1 phosphorylates CDC25C allowing its interaction with 14-3-3 and inhibition of its phosphatase activity [178,179]. CHK1 also phosphorylates WEE1, affecting its distribution through interaction with 14-3-3. Finally, CHK1 also phosphorylates CDC25A leading to CDK2 inactivation and delayed intra-S phase [180].

DNA damage response (DDR) to various types of DNA insults is a well orchestrated process and is required to maintain genomic integrity (Figure 1) [9-12]. In response to DSBs, a signaling process activates cell cycle checkpoints and pauses cell cycle progression, thus granting time for damaged cells to repair their DNA (Figure 2** and section 2s) **[13]. Two major repair pathways for DSBs exist in mammalian cells; the homologous recombination (HR) and the non-homologous end-joining (NHEJ) pathways [14]. The HR pathway is error free but requires an intact homologous template such as a sister chromatid. The NHEJ recombination pathway is the prominent pathway for DSB repair in mammalian cells; however this pathway is error prone as unlike HR pathway it does not require a long homologous sequence to guide the repair [15]. The choice of HR or NHEJ pathway for repairing DSBs is dependent on the phases of the cell cycle. HR is the main DSB repair pathway used during the S and G2 phase when sister chromatids are intact and readily available, whereas NHEJ is predominant during the G1 phase of the cell cycle [16,17].

In the situation where cells are unable to repair their DSBs, DDR pathways are activated by kinases such as ATM and ATR, thus leading to the senescence or the death of damaged cells [18-21] (Figure 2). Failure to detect, signal or repair DSBs, can result in damaged cells escaping the cell cycle checkpoints and evading death and thus these damaged cells can potentially generate a progeny that carries harmful mutations or chromosomal aberrations. Such genomic instability is a key driving force for the development of various human syndromes, immunodeficiency, aging and cancer [1,9].

During the past few years, significant progress has been made toward better understanding of the mechanisms underlying the DSB responses in mammalian cells. Current data have demonstrated the importance of the early detection and signaling of DSBs. This signaling of DSBs has emerged as a highly regulated and complex process. Mutations in a number of genes involved in DSBs signaling have highlighted the importance of this process.

In this review we will focus on proteins that have been demonstrated to play important roles in the detection and signaling of DSBs. We will discuss a number of post-translational modifications (PTMs) including phosphorylation, ubiquitylation, SUMOylation, acetylation and methylation, that are critical for DSBs signaling. Mutations in a number of genes involved in the signaling of DSBs have been demonstrated to lead to various human pathologies including cancer and will be also discussed in this review.

### Signaling of DNA Double-Strand Breaks

#### Role of Phosphorylation and Dephosphorylation in the Signaling of DSBs

**Figure 3:**
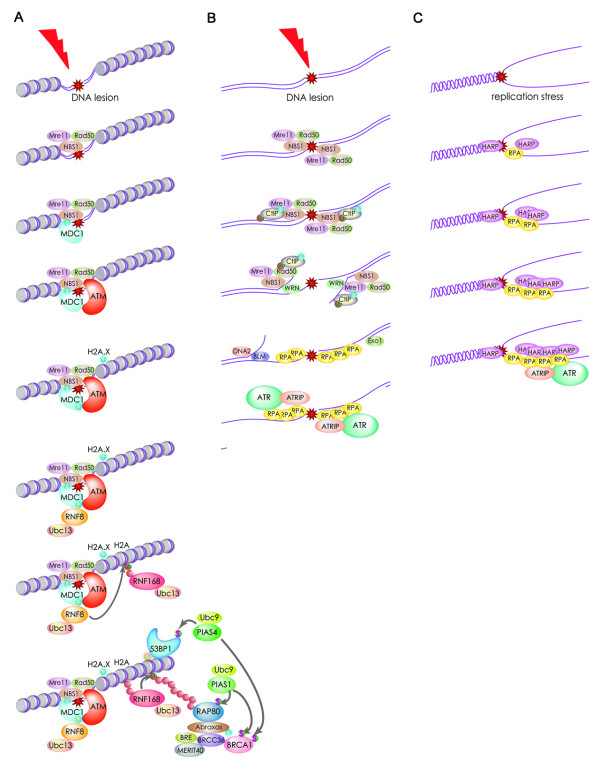
**Multistep signaling of DNA double-stranded breaks**. ATM and ATR, through the phosphorylation of their downstream substrates, play central roles in DSB signaling and the activation of cell cycle checkpoints. (**A**) Mre11/Rad50/NBS1 (MRN)-complex mainly recognizes DSBs and undergo ATM activation. Activated ATM phophorylates multiple substrates, including H2A.X and MDC1. MDC1 recognizes γ-H2A.X and bind to it. Then RNF8 recognizes the thyreonyl-phosphorylated MDC1 via its FHA domain and is recruited to DSB sites where it ubiquitylates the histone H2A. Subsequently, RNF168 is recruited to the ubiquitylated H2A, further ubiquitylating it with Ubc13 and likely leading to the modification of the chromatin structure. Through its tutor domain, 53BP1 is recruited to histone H4 di-methylated on lysine 20, while RAP80 and its protein complex are recruited to the polyubiquitylated H2A. (**B**) In the S and G2 phase, DSBs are recognized by the MRN-complex and CtIP is recruited to DSBs. CtIP is phosphorylated by CDK and ATM, and is ubiquitylated by BRCA1 in response to DNA damage. Collaboration of MRN and CtIP results in DSB end resection, and subsequently DNA2/BLM-complex and Exo1 promote further the resection of DSB ends leading to generation of single strand DNA (ssDNA). RPA binds to the ssDNA and induces ATR activation and HR repair [181-184]. (**C**) Replication fork stalling. The damaged single-stranded DNA is coated by RPA and this structure plays crucial roles in the recruitment of the helicase HARP and the ATR-ATRIP complex to sites of DNA damage[185-187]. P: phosphorylation, Ub: ubiquitin, S: SUMOylation, ATRIP: ATR interacting protein, RPA: Replication protein A. HARP: HepA-related protein.

Mammalian cells have evolved a sophisticated network of proteins to sense and signal DSBs (Figure 3). Members of the Phosphatidylinositol-3 kinase-related kinases (PIKK) family play important roles in different stages of DSB signaling through their ability to phosphorylate a number of substrates leading to the propagation of DSB signaling [22]. Members of the PIKK family consist of serine/threonine protein kinases with a conserved kinase domain (KD). PIKK family members also show conservation of three other domains that regulate their KD activity. These three domains are the FRAP-ATM-TRRAP (FAT) domain, the PIKK-regulatory domain (PRD) and the FRAP-ATM-TRRAP-C-terminal (FATC) domain [23-25]}. The FATC domain is critical for the kinase activity of the PIKK family members and mutations of this domain reduce the kinase activity of PIKK family members [23,26]. In addition, the FATC domain was shown to serve protein-protein interactions. The C-terminal PRD is located between the KD and FATC domains and is the target of posttranslational modifications.

Three members of the PIKK family are essential for the response to DSBs (Figure 2). These kinases are Ataxia-Telangiectasia-Mutated (ATM), Ataxia Telangiectasia and Rad3 Related (ATR) and DNA-dependent Protein Kinase catalytic subunit (DNA-PKcs). ATM and DNA-PKcs are critical for the signaling of DSBs while ATR is mainly involved in the response to DNA single strand breaks (SSBs) and stalled replication forks [13,22,27]. Remarkably, recent studies indicated that these PIKKs have a large number of substrates, and more than 900 sites of over 700 proteins have been identified as potential phosphorylation sites for ATM and ATR in response to DNA damage [28]. It has been shown that when ATM activity is compromised, DNA-PKcs can compensate to a certain degree for the defective ATM in some situations, such as V(D)J or class switch recombination in lymphocytes [29-31]. However, ATM, DNA-PKcs and ATR functions are all essential as demonstrated by the association of their mutations with a number of human syndromes discussed in section 3.

In mammalian cells, DSB ends are recognized by the MRE11/Rad50/NBS1 (MRN) and the KU70/KU80 complexes that function as sensors of DSBs but are also involved in processing the DNA break ends [32,33]. The MRN complex recruits ATM, while KU70/80 recruits DNA-PKcs, to DNA lesion sites [34]. The MRE11 (Meiotic Recombination 11) protein is conserved from archaea to mammalian cells and is involved in HR, NHEJ and maintenance of telomeres [35]. MRE11 has single strand endonuclease activity and 3' to 5' DNA exonuclease activity. RAD50 is a homolog of the *S. cerevisiae *rad50 and is a member of the structural maintenance of chromosome (SMC) protein family [36]. The third partner in the MRN complex is the NBS1 (Nijmegen breakage syndrome 1) protein also known as Nibrin and p95. Interaction of MRE11 with RAD50 upregulates both its exonuclease and endonuclease activities, while its interaction with NBS1 upregulates only its endonuclease activity [37].

The MRN complex interacts with the N-terminal domain of ATM and recruits it to DSBs. The MRN complex is also required for ATM activation[38-41]. In undamaged cells, ATM forms inactive dimers or multimers; however, upon the formation of DSBs, ATM is autophosphorylated on serine 1981 leading to its dissociation into active monomers [25,42]. In addition to serine 1981, ATM is also autophosphorylated on serine 367 and serine 1893 and mutations of these sites reduce its activity [25,42,43]. ATM substrates such as NBS1 are rapidly phosphorylated following ATM autophosphorylation and thus ATM autophosphorylation is considered as a hallmark for the activation of ATM [25,44]. Autophosphorylation of ATM serine 1981 has been demonstrated to be indispensable for its monomerization and chromatin association at the DSB sites [45]. A recent study demonstrated that ATM autophosphorylation is not required for its initial localization to DSBs, but is important its stabilization at the sites of DSBs [46]. The recent finding that transgenic mice carrying alanine substituted autophosphorylation serine sites 1987, 367 and 1899 of Atm (corresponding to human ATM serine 1981, 367 and 1893) display functional Atm-dependent responses have raised questions regarding the importance of ATM autophosphorylation for its *in vivo *functions [47].

Three serine/threonine protein phosphatase PP2A, PP5 and WIP1, have been implicated in the control of ATM activation [48-50]. In undamaged cells, PP2A interacts with ATM to ensure that ATM is not inappropriately activated by autophosphorylation [48]. In response to DNA DSBs, PP2A dissociates from ATM, therefore minimizing the risk of competition between phosphorylation and phosphatase activities [48]. The phosphatase WIP1 is also capable of removing phosphates from serine 367 and serine 1981 autophosphorylation sites [49], while PP5 has been shown to interact with ATM and to upregulate its activity in response to DNA damage [50].

In response to DNA damage, activated ATM, or alternatively DNA-PKcs or ATR, rapidly phosphorylates H2A.X, a histone H2A variant, on its serine 139 (γ-H2A.X) [51]. The large number of substrates for activated ATM includes proteins such as the structural maintenance of chromosome-1 (SMC1), NBS1, checkpoint kinase 2 (CHK2), tumor protein 53 (P53), breast cancer early onset-1 (BRCA1) and mediator of DNA damage checkpoint protein-1 (MDC1) [25].

Tyrosine 142 residue of H2A.X is constitutively phosphorylated and its subsequent dephosphorylation in response to DNA damage may enhance MDC1 and ATM recruitment to extend and maintain γ-H2A.X phosphorylation [52]. Phosphorylation and dephosphorylation of H2A.X on its tyrosine 142 is regulated by the tyrosine kinase WSTF (Williams-Beuren syndrome transcription factor, also known as BAZ1B) and the protein phosphatase EYA (Eyes Absent) [53,54]. Pre-existing H2A.X tyrosine 142 phosphorylation by WSTF is critical for DNA damage induced formation and retention of γ-H2A.X foci after DNA damage, while EYA removes the tyrosine phosphorylation in response to DNA damage. The presence of phosphorylated tyrosine 142 on H2A.X interferes with the MDC1 mediated recruitment of DNA damage signaling and repair factors to phosphorylated serine 139 residue of H2A.X[54].

The elimination of γ-H2A.X at DNA damage sites is required for appropriate DNA damage repair. In mammalian cells, dephosphorylation of serine 139 residue of H2A.X is regulated by the protein phosphatase PP2A and PP4 [55-57]. While PP4 dephosphorylates serine 139 of H2A.X in response to relatively low levels of DNA damage that occur during DNA replication, higher levels of damage might require both PP2A and PP4 to eliminate γ-H2AX [56]. In the absence of the catalytic subunit of PP2A (PP2Ac), γ-H2A.X foci persist and DNA repair is impaired [55]. The effect of PP2A on γ-H2A.X is independent of ATM, ATR and DNA-PKcs. PP4 dephosphorylates γ-H2A.X both at the sites of DNA damage and in undamaged chromatin, and depletion of PP4 catalytic subunit (PP4C) causes prolonged MDC1 retention at the sites of DNA lesions and results in a prolonged checkpoint arrest [57].

MDC1 recognizes γ-H2A.X and binds to it via its tandem BRCA1 C-terminal (BRCT) domains [39]. This recruitment of MDC1 to DSB sites further promotes the accumulation and retention of active ATM and MRN complexes to γ-H2A.X containing chromatin surrounding the sites of DNA damage [38,39,58,59]. The MDC1 serine-aspartic acid-threonine (SDT) repeats near its N-terminus, which are phosphorylated by Caseine Kinase 2 (CK2), interact with the N-terminal fork-head associated (FHA) domain of NBS1 [60-63]. In undamaged cells, MDC1 exists in a complex with MRN [60-63]; however, following ATM activation, MDC1 and its interacting MRN complex are recruited to γ-H2A.X [60-63]. Interestingly, while the N-terminus of NBS1 interacts with MDC1, its C-terminus interacts with ATM. Hence, MDC1 bridges the interaction of MRN to γ-H2A.X and ATM. The concentrated binding of ATM to MDC1 and MRN further promotes the phosphorylation of H2A.X and triggers the accumulation and retention at the sites of DNA lesion of a number of factors essential for DSB signaling including the RING finger proteins RNF8 and RNF168, p53 binding protein 1 (53BP1) and BRCA1 [64].

These examples of phosphorylation/dephosphorylation events highlight the importance of these processes in the signaling of DSBs.

#### Roles of Ubiquitylation and SUMOylation in the Signal Transduction of DSBs

Recent evidence have demonstrated major roles for ubiquitylation and Sumoylation in the signaling of DSBs [65-68]. Post-translational modifications of proteins by ubiquitin or small ubiquitin-like modifier (SUMO) are ATP-dependent and are mediated by a sequential enzyme cascade that involves ubiquitin or SUMO activating enzymes (E1), conjugation enzymes (E2) and protein ligases (E3) [69-71]. Ubiquitin is activated by the E1 enzyme and then a thioester linkage is formed between the carboxyl terminal glycine of ubiquitin and the reactive cysteine in E1. Subsequently, the ubiquitin is transferred from E1 to the active site cysteine of the conjugation enzyme E2 through a trans-thioesterification reaction. The E2 then interacts with its specific E3 ligase that catalyzes an isopeptide bond between an ε-amino group of a lysine residue in the substrate and the C-terminal glycine of ubiquitin or SUMO, thus completing the post-translational modification.

E3 ligases govern the specificity of ubiquitylated substrates and fall into four major groups; the HECT (Homologous to the E6-AP Carboxyl Terminus) type E3, the RING (Really Interesting New Gene) type E3, the U-box type E3 and the A20-type C2/C2 zinc-finger [72]. In the ubiquitin pathways, one E1 enzyme is shared by all ubiquitin ligases, and the combination of 20 to 30 E2 enzymes and thousands of E3 ligases provide the specificity of the ubiquitylated substrates [71]. Ubiquitin is highly conserved among eukaryotes, and possesses seven lysine (K) residues (K6, K11, K27, K29, K33, K48 and K63) that can be conjugated to other ubiquitins to generate polyubiquitin chains and sometimes mixed-linkage ubiquitin chains [73-76]. A number of ubiquitin modifications including monoubiquitylation, multiubiquitylation and polyubiquitylation serve diverse functions and cellular processes. Monoubiquitylation is involved in a wide variety of cellular processes including membrane protein transport, viral budding, protein localization and DNA damage responses [77-79]. While different types of ubiquitin linkages exist, K48 and K63-linked polyubiquitin chains are the best characterized. K48 mediated polyubiquitylation is known to target substrates for proteasomal degradation [80] whereas K63-linked polyubiquitylation is important for signal transduction during immune responses, cell cycle and DNA damage responses [81-84].

In the SUMOylation pathway, three mammalian SUMO isoforms can be used for covalent modifications of protein substrates. SUMO-1 conjugates only a single SUMO moiety to a protein substrate, while SUMO-2 and SUMO-3 which are similar, can form poly-SUMO chains. SUMO isoforms are initially cleaved by SUMO-specific proteases (SNEPs). Subsequently the cleaved SUMOs are activated by the E1 enzyme that consists of SUMO1 activating enzyme subunit (SAE) 1 and 2 also known as Uba2 and Aos1. SUMO is then transferred to the single E2 enzyme UBC9 and subsequently ligated to the target substrates by a few E3 ligases known as Protein Inhibitor of Activated STAT (PIAS1, 2,3 and 4) [71,85].

Similar to other cellular processes, different ubiquitin E3 ligases including RNF8, RNF168 and BRCA1/BARD1 have been demonstrated to play central roles in the early signaling of DSBs (Figure 3). RNF8 and RNF168 function in collaboration with the E2 enzyme UBC13 [84], while the ligase BRCA1/BARD1 requires the E2 enzyme Ubch5c for its functions [86].

In response to DSBs, the three conserved threonine/glutamine/X/phenylalanine (T-Q-X-F) clusters of MDC1 are phosphorylated by ATM [28,87-89]. The threonyl-phosphorylated MDC1 interacts with the FHA domain of the E3 ligase RNF8 and recruits it to DSB sites [87-89]. In addition to its N-terminal FHA domain, RNF8 also contains a C-terminal RING finger domain important for its E3 ligase activities. Once RNF8 is recruited by MDC1 to DSB sites, it collaborates with UBC13 to mediate K63-linked ubiquitylation of the histones H2A and H2A.X at the flanking sites of the DNA lesion [90]. The histone H2A has been previously demonstrated to be mono-ubiquitylated at K119 by the E3 ligases RING1B (RING2/RNF2), RING1A/RING1 and 2A-HUB/hRUL138 [91-95]. In addition, 5 to 15% of H2A is estimated to be constitutively ubiquitylated [96,97].

RNF8 ubiquitylated H2A serves to recruit the E3 ligase RNF168 through its Motif Interacting with Ubiquitin 2 (MIU2) [98-100]. RNF168 and UBC13 mediate K63-linked polyubiquitination of H2A and this polyubiquitylation has been proposed to modulate the chromatin structure facilitating the recruitment of 53BP1 to DSB sites. K63-linked polyubiquitylation of H2A also serves to recruit Receptor-Associated Protein 80 (RAP80) via its tandem Ubiquitin Interaction Motifs (UIMs) [101-105]. Subsequent to its interaction with ubiquitylated histones, RAP80 facilitates the recruitment to the DSB flanking sites of the BRCA1-A complex that in addition to RAP80, contains BRCA1, BARD1, ABRAXAS/FAM175A, BRCC3/BRCC36, BRE/BRCC45 and MERIT40/NBA1 [101].

Interestingly, the heterodimer BRCA1/BARD1 was reported to form K6-linked ubiquitin chain during DNA replication or repair [106,107]. BRCA1, in collaboration with BARD1, has been shown to mediate ubiquitylation of several substrates *in vitro*; however, it has been difficult to identify its *in vivo *ubiquitylation substrates. γ-tubulin [108], RPB8 [109], Topoisomerase IIα [110], and CtIP [111] are among the established *in vivo *ubiquitylation substrates for BRCA1.

While the importance of SUMOylation in DNA repair and replication has been demonstrated earlier [71,112-115], recent studies have highlighted its close relationship with ubiquitylation during DSB signaling [66,68]. The SUMO E3 ligases PIAS1 and PIAS4 were shown to be recruited to DSBs and to be required for the DSB-induced ubiquitylation mediated by RNF8 and RNF168. PIAS4 is required for the accumulation of RNF168 on DSB sites, through regulation of RNF8 and/or RNF168 E3 ligase activities or through amplification of protein-protein interactions. Furthermore, these studies demonstrated the SUMOylation of RAP80 and indicated that RNF168 SUMOylation is mediated by PIAS4 or PIAS1 [67,116]. Interestingly, in response to DSBs, PIAS4 also mediates SUMO1 modification of 53BP1 and BRCA1 while SUMO2/3 modification of BRCA1 is also mediated by PIAS1. These studies also indicated that SUMOylation of BRCA1 is critical for its ubiquitin E3 ligase activity [66].

Thus, in addition to phosphorylation, increasing evidence indicates the requirement for other posttranslational modifications including ubiquitylation and SUMOylation for the signaling of DSBs.

### DNA Damage Response Proteins and Hereditary Human Diseases

A number of hereditary human diseases/syndromes have been associated with mutations that target genes involved in the signaling of DSBs. Some of these genes and the human diseases and syndromes associated with their mutations are discussed here.

#### MRE11, RAD50 and NBS1

The MRN complex is recruited to the DNA damage site and activates ATM. In contrast to Atm, knockout mouse models for Mre11, Rad50 and Nbs1 are embryonic lethal [117-119]. Hypomorphic mutant mice for *Nbs1 *or *Mre11 *exhibit increased radiosensitivity, defective cell cycle checkpoints, chromosome instability and immunodeficiency [120-123]. In contrast to *Nbs1 *or *Mre11 *hypomorphic mutant mice, *Rad50 *hypomorphic mutants show partial embryonic lethality and exhibit progressive hematopoietic stem cell failure without increased radiosensitivity or defective cell cycle checkpoints [124].

In human, *NBS1 *mutations have been associated with the Nijmegen breakage syndrome (MIM #251260) characterized by microcephaly, radiosensitivity, growth delay, ovarian dysgenesis, immunodeficiency and marked cancer predisposition [125]. Patients with this syndrome are prone to infectious diseases owing to humoral and cellular immune deficiency. At least 10 different *NBS1 *mutations, most of them leading to truncated NBS1 proteins or amino-acid substitutions that might affect NBS1 protein-protein interactions, have been reported for patients with the Nijmegen breakage syndrome. These NBS1 mutants with truncations or amino-acid substitutions are likely to retain partial NBS1 activities, thus explaining the milder defects observed in these patients compared to *Nbs1 *null mutation in mice that leads to embryonic lethality [118,125].

Hypomorphic mutations of human *MRE11 *gene result in ataxia telangiectagia like disorder (ATLD; MIM #604391) characterized by cerebellar atrophy and radiosensitivity without marked immunodeficiency, cancer predisposition or telangiectagia [126]. ATLD has slower progression and is a milder condition compared to ataxia telangiectagia (A-T) syndrome that associates with *ATM *mutations. In contrast to NBS and A-T, patients with ATLD have almost normal immune responses. While the limited number of ATLD patients does not allow determination of whether this syndrome associates with increased cancer risk, *Mre^ATLD1/ATLD1 ^*mice exhibited impaired Atm functions but showed no increased cancer susceptibility [121].

RAD50 deficiency (MIM #613078) was reported for a patient with NBS-like disorder characterized by microcephaly, growth retardation and radiosensitivity without marked immunodeficiency or cancer predisposition [127]. Cells from this patient exhibit cellular radio sensitivity and defective activation of cell cycle checkpoints in response to DSBs.

#### ATM

ATM plays important roles in the responses to induced DSBs as well as to programmed DSBs generated during V(D)J and meiotic recombinations. Atm deficiency in mice results in defective activation of cell cycle checkpoints, impaired rearrangement and expression of T-cell receptors, reduced class switch recombination of immunoglobulins, sterility and tumorigenesis [22,128-133].

In human, mutations of *ATM *result in ataxia-telangiectagia (A-T; MIM #208900) characterized by progressive cerebellar ataxia, oculocutaneous telangiectagia, immune defects and lymphoid tumors [134]. In addition, primary immunodeficiency, radiosensitivity and progressive neurodegeneration are hallmarks of patients with A-T. The cause of neurodegeneration in A-T patients remains unknown. While, one-third of A-T patients develop malignancies including lymphoid and breast cancers, some A-T patients also develop insulin-resistant diabetes and metabolic syndromes [25,135-137].

Somatic mutations of *ATM *have been also associated with human cancers. Analysis of coding exons of 518 protein kinases in 210 different human cancers, indicated that *ATM *gene ranked third in terms of mutation frequency, behind *Titin *and *BRAF *(B-Raf proto-oncogene serine/threonine-protein kinase) [138].

#### ATR

In humans, the autosomal recessive disorder known as Seckel syndrome (MIM #210600) is associated with *ATR *mutation [139,140]. Features of this syndrome include microcephaly, developmental delay, mental retardation, mild sensitivity to ultraviolet radiation, characteristic facial features but no marked radiosensitivity and immunodeficiency. ATR is a phosphoinositol 3-kinase-like kinase that is activated by single-strand regions of DNA. Several proteins are required for the ATR-signaling response and defects of these proteins can result in Seckel-like clinical features [141]. Thus, haploinsufficiency of replication protein A1 (*RPA1*) and replication factor C2 (*RFC2*) has been associated with the Miller-Dieker lissencephaly syndrome (MIM #247200) and the William-Beuren syndrome (MIM #194050), respectively. These syndromes are also characterized by microcephaly, growth retardation and facial abnormality [142,143].

In contrast to *Atm*, null *Atr *mutation leads to early embryonic lethality in mice [144]. Interestingly, a mouse model carrying the *ATR *mutation (A2101G) associated with the Seckel syndrome in patients, recapitulated the symptoms of the human disease [145].

#### DNA-PKcs

*DNA-PKcs*-null mice are viable but exhibit radiosensitivity, immunodeficiency, and hyperplasia and dysplasia of the intestinal mucosa. However, these mutant mice are not growth retarded [146].

In humans, *DNA-PKcs *mutations result in radiosensitive severe combined immunodeficiency (RS-SCID) characterized by radiosensitivity and immunodeficiency without microcephaly and mental retardation [147]. The B-cell development defects of patients with this RS-SCID are reminiscent of Artemis deficient SCID patients and RAG (recombination activating genes) deficient SCID patients [148,149]. No increased cancer predisposition has been reported for patients with the RS-SCID associated with *DNA-PKcs *mutations.

#### RNF168

The recently identified RIDDLE syndrome (MIM #611943) has been associated with homozygous *RNF168 *mutations [99,150]. This syndrome is very rare as only one patient has been identified to date. Characteristics of the RIDDLE syndrome include radiosensitivity, immunodeficiency, dysmorphic features and learning difficulties. The identified RIDDLE patient has been reported to have low concentrations of serum immunoglobulin while the number of his peripheral lymphocytes remained within normal ranges. Analysis of skin fibroblasts from this patient showed a mildly increased radiosensitivity, defective intra-S and G2/M phase checkpoints, and impaired recruitment of 53BP1, RAP80 and BRCA1 to DSB sites. More patients with the RIDDLE syndrome are needed to determine the spectrum of pathologies associated with this syndrome and whether it is associated with increased cancer predisposition.

Mouse models carrying mutations of *Rnf8*, an E3 ligase required for the recruitment of Rnf168 to sites of DNA damage, have been reported. While no human syndrome/disease have been reported yet to associate with *RNF8 *mutations, *Rnf8^-/- ^*mice, similar to the RIDDLE syndrome patient, suffer from defective IgH Class Switch Recombination and are immunodeficient [151,152]. *Rnf8^-/- ^*males also exhibit impaired spermatogenesis and are infertile [151-153]. Remarkably, *Rnf8^-/- ^*mice are growth retarded and display increased cancer predisposition, demonstrating that Rnf8 is a novel tumor suppressor [151]. Studies of mouse models for *Rnf168 *mutations are required to determine whether they reproduce characteristics of the RIDDLE syndrome and whether, similar to Rnf8, Rnf168 plays a role in cancer.

#### BRCA1

BRCA1 is important for a number of cellular functions including activation of cell cycle checkpoints and repair of DSBs through HR pathway [154]. Other functions that have been ascribed to BRCA1 include transcription, ubiquitylation, estrogen receptor signaling, and chromatin remodeling [155-160].

Mutations of the tumor suppressor BRCA1 (MIM #113705) predispose women for breast and ovarian cancer [161,162]. Typically, a germline mutation in one of the *BRCA1 *alleles is inherited from one of the parents and loss of heterozygosity (LOH) of the Wild-type *BRCA1 *allele is required in order for tumors to develop. Women with inherited inactivating mutation of *BRCA1 *gene have about a 65-80% lifetime risk of developing breast cancer [163]. The familial breast cancer associated with BRCA1 inactivation develops at younger ages and is frequently bilateral compared to the sporadic types of breast cancer. In addition, women with inherited mutations of *BRCA1 *gene are also predisposed for ovarian cancer with a lifetime risk about 37-62% compared to less than 2% for women that do not carry *BRCA1 *or *BRCA2 *mutations [163]. BRCA1 forms an E3 ligase with its partner BARD1 and its RING finger domain is essential for this E3 ligase activity. Mutation of cysteine 61 in the RING finger domain of BRCA1 (BRCA1C61G) has been observed in patients with the familial breast cancer.

A remarkable therapeutic opportunity has been discovered recently for cancer patients that carry mutations of the familial breast cancer genes *BRCA1 *or *BRCA2*. Both genes play important roles in the HR repair pathway. Inactivation of either BRCA1 or BRCA2 has been shown to drastically decreases HR activities [164,165]. Interestingly, recent studies indicated that inhibition of the poly (ADP-ribose) polymerase (PARP) leads to synthetic lethality of tumors deficient for either BRCA1 or BRCA2 [166-169]. PARP1 and PARP2 facilitate the repair of SSBs by recruiting DNA repair proteins to the damaged sites and promoting the restart of stalled replication forks [170]. In the absence of PARP activity, SSBs can be converted to DSBs during replication. As BRCA1 and BRCA2 deficient tumors are defective in HR mediated repair of DSBs, inactivation of PARP in these tumors leads to collapsed replication folks, generation of DSBs, cell cycle arrest and cell death [166,167]. Several PARP inhibitors, including the third generation PARP inhibitors with high potency and specificity, are in clinical trial development worldwide [170]. There is currently great hope that these PARP inhibitors may represent a great opportunity to improve the therapy for BRCA1 and BRCA2 cancer patients.

Several mouse models for *Brca1 *mutation have been reported[171]. While *Brca1 *null mutations lead to early embryonic lethality [172], targeted *Brca1 *mutations to mammary epithelial cells lead to mammary tumorigenesis [173,174]. In addition, inactivation of the Chk2-p53 pathway was found to synergize the development of mammary tumors associated with Brca1 deficiency [174].

Interestingly, recent studies demonstrated that inactivation of 53bp1 rescued the proliferation defect and hypersensitivity to DNA damaging agents, and partially restored the HR defects of Brca1 deficient cells [175,176]. Inactivation of 53bp1 suppressed the development of mammary tumors in mice carrying hypomorphic *Brca1 *mutations (*Brca1^Δ11/Δ11^*) [175]. 53BP1 expression was recently found frequently decreased in a subset of basal-like/triple-negative breast cancers and in BRCA1 or BRCA2 negative breast tumors [176]. Further investigations are needed to clarify the precise effects of 53BP1 inactivation on the development of *BRCA1 *associated breast cancers, the responses of these cancers to therapies and the mechanisms and significance of the reduced 53BP1 expression in breast tumors with *BRCA1 *or *BRCA2 *mutations.

## Conclusion

Remarkable progress has been made over the past few years regarding the signaling and repair mechanisms of DNA double-strand breaks. Post-translational modifications have emerged as key factors that regulate these processes. Although more studies are required to identify missing players in these repair/signaling pathways and the mechanisms that control these processes, it is likely that the considerable amount of knowledge accumulated over the years will facilitate the development of better therapies for human diseases including cancer.

## Competing interests

The authors declare that they have no competing interests.

## Authors' contributions

TB, MB and RH wrote the manuscript. All authors read and approved the final manuscript.
